# Inflammation in multiple system atrophy

**DOI:** 10.3389/fimmu.2023.1214677

**Published:** 2023-06-22

**Authors:** Marta Leńska-Mieciek, Natalia Madetko-Alster, Piotr Alster, Leszek Królicki, Urszula Fiszer, Dariusz Koziorowski

**Affiliations:** ^1^ Department of Neurology and Epileptology, Centre of Postgraduate Medical Education, Warsaw, Poland; ^2^ Department of Neurology, Medical University of Warsaw, Warsaw, Poland; ^3^ Department of Nuclear Medicine, Medical University of Warsaw, Warsaw, Poland

**Keywords:** multiple system atrophy (MSA), neurodegeneration, inflammation, synucleinopathies, tauopathies

## Abstract

Misfolding protein aggregation inside or outside cells is the major pathological hallmark of several neurodegenerative diseases. Among proteinopathies are neurodegenerative diseases with atypical Parkinsonism and an accumulation of insoluble fibrillary alpha-synuclein (synucleinopathies) or hyperphosphorylated tau protein fragments (tauopathies). As there are no therapies available to slow or halt the progression of these disea ses, targeting the inflammatory process is a promising approach. The inflammatory biomarkers could also help in the differential diagnosis of Parkinsonian syndromes. Here, we review inflammation’s role in multiple systems atrophy pathogenesis, diagnosis, and treatment.

## 1 Introduction

Multiple system atrophy (MSA) is a rare, progressive proteinopathy with an accumulation of insoluble fibrillary alpha-synuclein (aSyn) that presents with various combinations of atypical Parkinsonian syndrome, autonomic failure, and cerebellar syndrome.

Most studies assessing the pathogenesis of MSA have focused on the mechanisms of aSyn accumulation in neurons and oligodendrocytes. The glial cytoplasmic inclusions (GCIs) in the oligodendrocytes are unique among synucleinopathies (1, 2).

## 2 Inflammation: effect or cause of neurodegeneration

**Figure 1:**
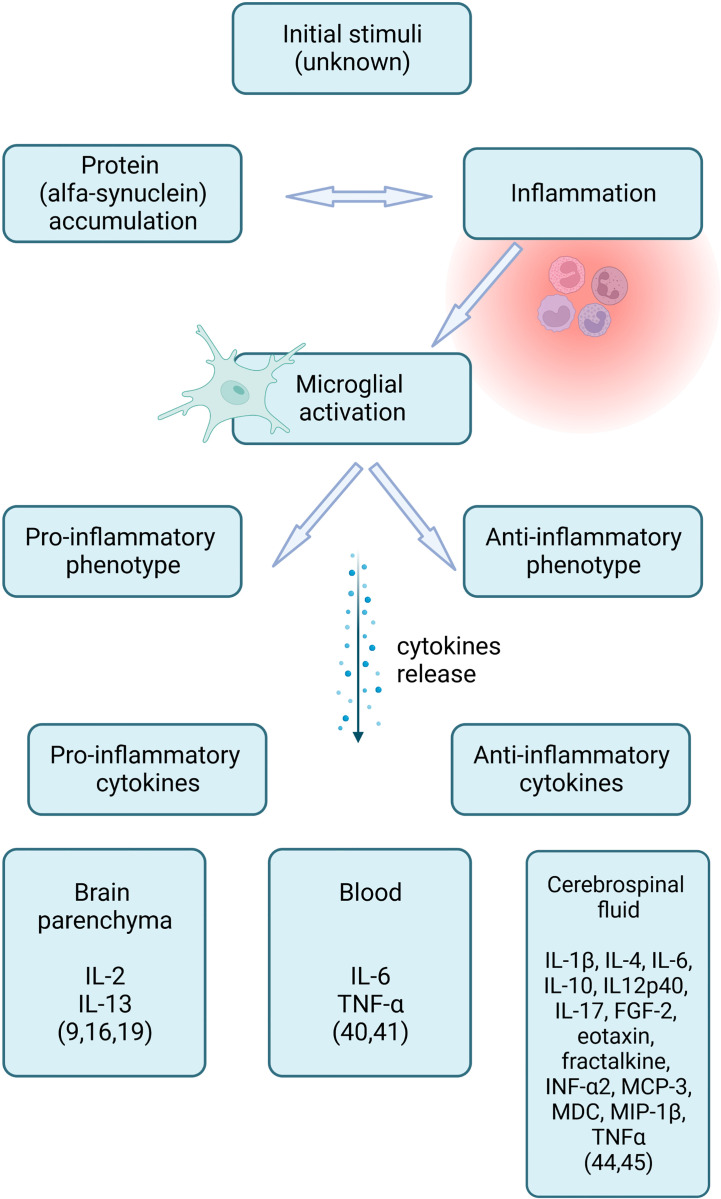
Inflammation and inflammatory cytokines in multiple system atrophy (created with BioRender.com).

Marked neuroinflammation is a suggested mechanism in MSA pathogenesis (3, 4). It remains uncertain if inflammation is a primary driver of protein accumulation, a response to the degeneration of neural tissue, or an aggravator of the process (5, 6). Increasing evidence suggests that misfolded aSyn triggers microglial activation and astrogliosis, which are thought to accelerate aSyn aggregation and oligodendroglial apoptosis (7–11). Although the initial stimulus in the MSA remains unknown, the inflammation mechanism is common (12). Extensive microglial activation and neuroinflammation with diffuse T-cell infiltration have been found in postmortem studies of MSA brains (8, 13–18). Activated microglia can either take up a pro-inflammatory or an anti-inflammatory phenotype. Depending on the type of activation, microglia release specific inflammatory cytokines, which can be detected in blood, cerebrospinal fluid (CSF), and the brain parenchyma (9) (**Figure 1**).

## 3 Immune response in MSA brain parenchyma

Brain inflammatory responses can enhance neuronal excitability, injure cells and increase blood-brain barrier permeability (12). The increased microglia antigen presentation (MHC class II+cells) indicates a reactive immune response (9). CD3+, CD4+, CD8+T cells, and an increase in pro-inflammatory cytokines (IL-2, IL-13) were found in the MSA brain (9, 16, 19). Increased density of interferon stimulator genes, (STING)- and TANK-binding kinase 1 (TBK1)-immunopositive astrocytes, was reported. Continuous STING activation is assumed to contribute to chronic inflammation (20).

Recent studies have shown that innate inflammatory processes cause the deteriorating course of chronic neurodegenerative diseases. Williams et al. proposed a hypothesis of inflammation in a mouse model of MSA, in which the accumulation of aSyn in oligodendrocytes leads to the upregulation of major histocompatibility class II antigen (MCHII) on microglia and promotes the infiltration of the CD4+T cells and monocytes from the blood. All pro-inflammatory cells promote the dysfunction of oligodendrocytes (19).

## 4 Inflammation-related gene expression

Changes in inflammation-related gene expression occur in the MSA brain, through both down- and up-regulation (9, 21, 22).

Reported changes in DNA methylation or the expression level of genes being more highly expressed in microglia/macrophages support the involvement of inflammatory processes in MSA (17, 23). The dysregulation of inflammation-related intracellular pathways results in aberrant mRNA levels (24).

Gene encoding inflammatory mediators are also an area of interest (11). Selected interleukins (IL-1A, IL-1B, IL-8) and TNF-α genetic polymorphism increased the risk of MSA, as well as variants of Triggering Receptor Expressed on Myeloid Cells 2 (TREM2) and nucleotide-binding oligomerisation domain containing 2 (NOD2) (25–29). Alpha-1-antichymotrypsin (ACT) gene polymorphism is a risk and modulating factor for MSA. Onset was earlier and the disease progressed faster in patients with the ACT-AA genotype (30).

An altered microRNA (miRNA) expression is associated with initiating and/or sustaining neuroinflammation. An increased brain expression of 214 miRNA was found in MSA, and of these dysregulated miRNA 83 were disease-specific (31). Abnormal levels of more than 60 miRNAs were found in the striatum of MSA patients, with miR-124-3p, miR-19a-3p, miR-27b-3p, and miR29c-3p as altered key regulators, mainly contributing to neuroinflammation (32).

Altered expression of miRNA was also found in the blood and plasma of MSA patients, both under- and over-expression. The results of the studies are divergent (33–35).

A study of CSF showed a difference between MSA and controls in four miRNAs (miR-19a, miR-19b, miR-24, miR34c) (36).

## 5 Activation of inflammatory pathways in MSA

### 5.1 Toll-like receptors signaling - pattern recognition receptor activation

TLRs have altered expression in the substantia nigra, the striatum, the cerebral cortex, and the nucleus dentatus (37). aSyn real-time quaking-induced conversion (RT-QuIC) products generated by the olfactory mucosa of MSA patients increase the transcription level of several inflammatory mediators, including TLR2 and tumour necrosis factor (TNF) receptor-associated factor 6 (TRAF6) (38). Preformed α-syn fibrils (PFF) increase the association between TLR2 and innate immune signal transduction adaptor MyD88, resulting in microglial activation (39).

### 5.2 Cytokines in blood and CSF

IL-6 and TNF-α serum levels are increased in MSA (40, 41). Levels of serum inflammatory markers might depend on the disease duration. Kim et al. reported no difference between MSA and controls in serum levels of IL-1β, IL-2, IL-6, IL-10, TNF-α, or high‐sensitivity C reactive protein (CRP). The authors postulated that peripheral inflammation might be vital to the initial pathogenesis of MSA (42).

Although the results are inconsistent, several cytokines and acute phase response (APR) protein levels altered in CSF. The levels of cytokines in MSA were similar to, or less than, the control (43). Compta et al. reported that 12 of the 38 tested cytokines were significantly increased (FGF-2, eotaxin, fractalkine, INF-α2, IL-10, MCP-3, IL12p40, MDC, IL-17, IL-6, MIP-1β, and TNFα) (44). Starhof et al. reported elevated CSF concentration of TFN-α, IL-1β, IL-4 and IL-6 (45).

### 5.3 Other peripheral inflammatory biomarkers

Some peripheral inflammatory biomarkers are elevated in MSA CSF: CRP, human serum amyloid A (SAA), ceruloplasmin, α1-antichymotrypsin, ferritin, and transthyretin (45–47). Folke et al. reported that anti-aSyn IgM nAbs plasma and CSF levels were reduced, and anti-aSyn IgG1, IgG2, and IgG3 CSF levels increased in MSA (48).

Higher serum monocyte to high-density lipoprotein ratio (MHR), neutrophil-to-lymphocyte ratio (NLR), and red cell distribution width to platelet ratio (RPR) was reported (49). An increase in CD3+ and CD4+ T-lymphocyte, the CD4+/CD8+ ratio, and a decrease in Ig concentration were found, especially in female patients (22).

## 6 Gut microbiome in MSA

Studies demonstrated altered gut microbiome composition and function with the presence of “pro-inflammatory” bacteria. The bacterial alterations differed between the studies and the studied populations (50). Engen et al. showed intestinal barrier dysfunction with a high relative abundance of gram-negative, putative “pro-inflammatory” bacteria. Patients had evidence of disrupted Zonula Occludens-1 (51). Some gut inflammation risk genes (LRRK2, NOD2) could also increase the risk of MSA, and “pro-inflammatory” bacteria may participate in stimulating MSA pathology (28, 52, 53).

## 7 Inflammation as a biomarker for MSA diagnosis

CSF inflammatory proteins (DNER, β-NGF) were reported as potential biomarkers, which could differentiate MSA from Parkinson’s Disease (PD) and controls. Both were down-regulated in MSA (54). CSF levels of TFN-α, IL-1β, IL-4, and IL-6 were significantly higher in MSA than in PD, and the best diagnostic discrimination was found with a combination of analytes (45).

MHR serum level was higher in MSA than in PD (49). The diagnostic value of serum miR-30c-5p in discriminating between MSA and PD was reported with a sensitivity of 82% and a specificity of 54% (55).

Two cytokines (MCP-3, MDC) were reported to discriminate MSA from non-MSA patients. In a respective binary logistic regression model combining age at inclusion and both these cytokines, younger age and increasing CSF levels of cytokines were predictors of MSA (44).

Ferritin and transthyretin CSF levels discriminated MSA patients from PD, progressive supranuclear palsy (PSP), and dementia with Lewy bodies (DLB) (47).

## 8 Inflammation as a biomarker for MSA progression

The more robust responses in the experimental viral oligodendroglial MSA model than in the PD model suggest that inflammation is responsible for the aggressive MSA clinical course (6). CSF CRP and IL-8 correlated with disease severity (46). Up-regulated serum miR-30c-5p levels are associated with disease duration (55). Recently CRP serum level was proposed as a prognostic biomarker related to higher mortality in MSA (56).

## 9 Challenges for future treatment

To date, there are no available therapies able to modify the course of MSA. Therefore, inflammation as a potential therapeutic target has been intensively studied (**Table 1**).

**Table 1:** Studies on potential anti-inflammatory therapies in MSA mice models and MSA patients.

MSA mice model		
Substance	Result	References
CSF1R selective inhibitor (PLX5622)	reduction of inflammation, an extension of lifespan	(57)
FTY720-Mitoxy	suppression of microglial activation	(58)
MPO inhibition	improvement of motor deficits	(59, 60)
minocycline	suppression of microglial activation	(61)
wtTIDM and wtNBD	suppression of microglial activation	(39)
MSA patients		
Substance	Result	References
NSAID	reduction of the disease risk	(62)
SSRI	disease-modification	(63, 64)
BHV-3241 (verdiperstat)	clinical trial – failed (phase III)	(10, 65)
minocycline	clinical trial – failed (phase II)	(66)
G-CSF cytokine	inconclusive results	(67)
IVIG	clinical improvement	(68)

CSF1R, colony-stimulating factor 1 receptor; IVIG, intravenous immunoglobulin; MPO, myeloperoxidase; MSA, multiple system atrophy; NSAID, non-aspirin non-steroidal anti-inflammatory drugs; SSRI, selective serotonin reuptake inhibitors; tNBD, wild-type of NEMO-biding domain; wtTIDM, wild-type toll like receptor 2 interaction domain of MyD88.

Repurposing existing drugs is one idea. Starhof et al. studied the use of anti-inflammatory drugs and the risk of MSA in a register-based case-control study. They observed a link between using non-aspirin non-steroidal anti-inflammatory drugs (NSAID) and the associated reduced risk of MSA (62).

Intriguingly, selective serotonin reuptake inhibitors may be disease-modifying, reducing inflammation-mediated neurodegenerative processes (63, 64).

Reduction of microglial activation may be the next promising future strategy for disease course modification.

Myeloperoxidase (MPO) inhibition in MSA mice in the early stage of the disease improves motor deficits more than in the advanced disease model (59, 60). This suggests time’s importance in MSA therapy when targeting microglial activation (69). The MPO inhibition was targeted in Phase III clinical trial with BHV-3241(verdiperstat). It was recently finished and reported that it failed to meet primary and key secondary endpoints (10, 65).

Suppression of microglial activation by early long-term minocycline treatment and protected dopaminergic neurons of the substantia nigra pars compacta in a transgenic mouse model was reported (61). A phase II randomised controlled trial failed to demonstrate motor improvement or neuroprotective effects (66).

Microglial development, survival, and maintenance depend on the colony-stimulating factor 1 receptor (CSF1R) (70). CSF1R selective inhibitor - PLX5622, which is available orally and crosses the blood-brain barrier, was administered to MSA mice. Reduced inflammatory signals, an extended lifespan, and delayed onset of neurologic symptoms were observed. Converse aggravation of motor deficits and alternation in neuronal and synaptic regulation in MBP29-hα-syn mice were reported. PLX5622 had no restorative effect on oligodendropathy (57).

Dutta et al. reported the intranasal administration of two peptides in preformed fibrils (PFF)-seeded mice. The wild-type TLR2-interaction domain of MyD88 (wtTIDM) peptide inhibits TLR2 activation, and the wild-type of NEMO-biding domain (wtNBD) peptide inhibits TLR2-mediated NF-κB activation. Disruption of TLR2-MyD88 interaction led to attenuation of microglial activation. Therefore wtTIDM and wtNBD might be the next therapeutic targets (39).

Inflammasomes are innate immune system receptors and sensors that regulate the activation of caspase-1 and induce inflammation. The regulation of microglial NLRP3-inflammasome activation was proposed as a potential therapeutic target (16).

In the mouse model, FTY720-Mitoxy reduces microglial activation in the cerebellum and has potent anti-inflammatory effects *in vivo* (58).

Both anti- and pro-inflammatory cytokines may be promising targets for potential MSA therapy. An anti-inflammatory cytokine, G-CSF induces neurogenesis and counteracts apoptosis. In MSA, a lower level of G-CSF was found. G-CSF was administrated to MSA patients subcutaneously in a minor clinical trial with inconclusive results (67).

The viral oligodendroglial MSA model has significant T-cell infiltration before any cell loss. That suggests the next therapeutic target, the blockade of T-cell infiltration (6, 19).

Intravenous immunoglobulin (IVIG) use in MSA patients was studied by Novak et al. in a small group of 7 patients. Unified Multiple System Atrophy Rating Scale (UMSARS) was improved in all, and UMSARS part II in 5 subjects (68). IgM levels gradually decrease during the disease course. This indicates that immunotherapy should start at the early stage of the disease (22).

Recent failures of single-drug therapies resulted in studies combining therapies targeting different aspects of the disease, with better results than each treatment alone (71).

## 10 Imaging inflammation in the brain

The main molecular targets in functional imaging of inflammatory processes are microglial cells (72–74) and the translocator protein (TSPO). TSPO (18 kDa) is a well-preserved and tryptophan-rich 169-amino-acid protein located on the outer side of the mitochondrial membrane. A characteristic feature of this protein is its ability to bind to benzodiazepines (72). The upregulation of TSPO is well documented in inflammatory processes.

Several radiopharmaceuticals have been developed that allow the assessment of TSPO expression using both Single-photon Emission Computed Tomography (SPECT) and positron emission tomography (PET) techniques. There are currently three generations of radiopharmaceuticals (75).

The first generation includes benzodiazepines and isoquinoline. [11C]Ro5-4864 belongs to the 4-chlorodiazepam family. It shows good binding affinity (Ki = 6 nM) for TSPO. The radiopharmaceutical is labeled with the C-11 isotope and can be used in the PET technique. This was the first tracer that specifically bound to TSPO but did not bind to the benzodiazepine subunit of the GABA receptor. Another radiopharmaceutical used is isoquinoline ([11C]-PK-11195). Isoquinoline binds to activated microglial cells and is an indicator of glial and macrophage activation.

The second generation belongs to different groups of chemical compounds. It is characterised by a much higher binding specificity to the TPSO protein and, thus a better signal-to-noise ratio (76, 77). An example is Phenoxy-phenylacetamide [11C]DAA1106 or [18F]DAA1106. However, these radiopharmaceuticals bind to microglial cells, astrocytes, and other cellular response cells. This ability to label other inflammatory cells limits the radiopharmaceutical use in studies on the role of microglial cells.

The third generation focused on the imaging of TSPO activity has almost eliminated the shortcomings of the existing tracers. They allow for better and more specific imaging of the activated microglia. Among others, this group includes tricyclic radiotracers like [18F]GE180, [11C]vinpocetine, or [18F]FEBMP and modified radiopharmaceuticals that had been developed previously: [18F]FE-DAA1106, [11C]DPA-713, [18F]DPA-714, [18F]PBR28, [18F]PBR111, [11C]SSR18075, [11C]CLINME, [123I]CLINDE (78–82).

Molecular imaging of the inflammatory processes is one of the essential tools in both research and clinical practice. Further progress in this field will allow us to approximate the pathomechanisms of several neurodegenerative diseases.

## 11 Inflammation in other atypical parkinsonism – tauopathies: similarities and differences

Inflammation in tauopathies is interpreted as a feature possibly coexisting with tau. However, additional mechanisms may interfere with the inflammatory-neurodegenerative relationship (83). The pathological protein tau in Alzheimer’s Disease (AD), corticobasal syndrome (CBS), and frontotemporal dementia (FTD) are considered initiatory points of inflammation leading to neurodegeneration (84). The accumulation of phosphorylated-tau is thought to be present before microglial activation (85). The activated microglia are observed around neurofibrillary tangles, which were described in the assessment with benzodiazepine receptor radiotracer in PET– [11C]PK11195 (86).

It was found that levels of double-stranded RNA are increased in astrocytes in PSP and AD (87).

Inflammatory genetic risk factors overlap between PD, AD, and FTD, but microglial-activated mechanisms may differ (88). The issue concerning inflammatory mechanisms in tauopathic atypical parkinsonism has not been widely described in the context of PSP. Some studies indicate oxidative stress as a likely significant feature in this context. One of the studies reported protein kinase p38SAPK as possibly affecting oxidative stress (89). In PSP, systemic inflammation is associated with cognitive decline (90). Information concerning CBS is relatively poor. It is affected by the lower incidence of the disease and more diverse pathologies, among which are PSP, CBD, FTD, AD, and, less commonly, vascular injury (91).

The assessments of biochemical and laboratory features of tauopathic parkinsonian syndromes in contemporary literature provide moderately encouraging results in the context of differentiation. Neurofilament light chains, linked with inflammatory-driven neurodegeneration, are increased in CBS and other atypical parkinsonisms, including PSP and MSA, compared to PD (92). Recently a study evaluating inflammatory parameters in Parkinsonian syndromes revealed increased levels of haptoglobin in PSP and ferritin and transthyretin in MSA. The study did not show the significance of these parameters in CBS (47). In PSP and CBS, the possible overlaps in clinical manifestation result in the need to verify whether adequate links can be observed in the context of pathophysiological features. Recent work based on the evaluation of the easily accessible peripheral NLR parameter did not reveal significant differences between PSP and CBS (93). On the other hand, the same parameter showed significantly increased values when compared to PD (94). The peripheral stimulation of the inflammatory mechanism in these diseases may be partly explained by dyslipidemia, vascular risk factors, and oxidative stress, which are related to the pathomechanisms leading to the diseases (91, 95). The abnormalities of inflammatory factors were evaluated in various Parkinsonian syndromes. However, the data suggest that the deviations may not strictly correlate with the pathology. The evaluations show significantly increased levels of CRP, TNF-alpha, IL-1-beta, IL-4, and IL-6 in comparing PSP and PD and between MSA and PD (45). Evaluations based on the analysis of the CSF revealed a pronounced increase of microglial-derived cytokines in PSP (46). The search for links between inflammatory pathways and the risk of PSP was also explored in the context of genes affecting microglial activation – chemokine receptor type 4 (96). Similar observations were made in the context of CBS and FTD.

The links between inflammation, PSP, and CBS were also verified in the context of their correlations with infection. Recently it was described in the context of neurosyphilis. The patient showed clinical manifestation of PSP with a moderate response to antibacterial treatment and a lack of improvement based on levodopa therapy (97). Several previous papers identified cases of clinical manifestations of syphilis as PSP or CBS. They were generally based on single cases and did not provide an overview of the possible mechanism leading to clinical manifestations of tauopathic parkinsonian syndromes (98, 99). A case of progressive supranuclear palsy-like phenotype as one of the primary symptoms of HIV is noteworthy among other instances of PSP possibly related to prior infection (100).

The possible inflammatory aspect of PSP is known in the context of microbiota, as diverse profiles in parkinsonisms may be interpreted as a consequence of microbial communities. In PSP, increased levels of Lactobacillaceae were found, interestingly similar to MSA (101). The possible impact of neuroinflammatory parameters in PSP is a developed feature in pharmacotherapy, as faecal microbiota transplantation was found to improve motor and non-motor elements of clinical manifestation in PSP-Richardson Syndromes (102). The anti-inflammatory impact was also assessed in the context of NSAID. No association between the use of NSAID and the incidence of PSP was revealed however, the study was affected by several limitations: a low number of examined patients and the use of NSAID was verified by phone. Additionally, the criteria of diagnosis used in this study were not contemporary (103, 104). None of the studies indicated the possible significance of differences in inflammatory stimulation depending on the phenotype of PSP. Moreover, the potential anti-inflammatory impact of future drugs in PSP seems likely to be achievable mainly in the preclinical or early stages of the disease. This leads to the need to develop practical methods enabling earlier diagnosis of PSP than is currently possible.

## 12 Conclusion and remarks

Neuroinflammation is a proposed mechanism in the pathogenesis of MSA and other neurodegenerative diseases. It remains uncertain if inflammation is a primary driver, a response to degeneration, or aggravates the process. Inflammatory biomarkers are potential markers for MSA diagnosis and progression, and inflammation is a potential therapeutic target for disease course modification.

## Author contributions

ML-M designed and reviewed the literature, adjusted the structure of the article, wrote drafts of the manuscript, supervised the work, and accepted the final version. PA reviewed the literature, wrote drafts of the manuscript, and accepted the final version. NM-A reviewed the literature, wrote drafts of the manuscript, and accepted the final version. UF supervision and final version acceptance. DK supervision and final version acceptance. LK reviewed the literature, wrote manuscript drafts, supervision, and final version acceptance. All authors contributed to the article and approved the submitted version.
